# Overexpression of protein regulator of cytokinesis 1 facilitates tumor growth and indicates unfavorable prognosis of patients with colon cancer

**DOI:** 10.1186/s12935-020-01618-9

**Published:** 2020-10-31

**Authors:** Tianxiang Xu, Xiaoxia Wang, Xiangdong Jia, Weishi Gao, Junhua Li, Fengying Gao, Ping Zhan, Wu Ji

**Affiliations:** 1 Center of Tumor, Inner Mongolia People’s Hospital, Hohhot, 010017 Inner Mongolia Autonomous Region China; 2 Intensive Care Unit, Inner Mongolia People’s Hospital, Hohhot, 010017 Inner Mongolia Autonomous Region China; 3 Department of Respiratory Medicine, Jinling Hospital, 305 East Zhongshan Road, Nanjing, 210002 Jiangsu China; 4 Department of General Surgery, Jinling Hospital, Southern Medical University , Nanjing, 210002 Jiangsu China

**Keywords:** Protein regulator of cytokinesis 1, Colon cancer, Prognosis, Proliferation, Apoptosis

## Abstract

**Background:**

Protein regulator of cytokinesis 1 (PRC1) has been reported to play important role in the pathogenesis of various cancers. However, its role in colon cancer has not been studied. Here, we aimed to investigate the biological functions and potential mechanism of PRC1 in colon cancer.

**Methods:**

The expression level of PRC1 in colon cancer tissues and cell lines was detected by quantitative real-time polymerase chain reaction (qRT-PCR), Western blotting, and immunohistochemical (IHC) staining of a tissue microarray (TMA). Furthermore, colon cancer cell lines HCT116 and SW480 were treated with short hairpin RNAs against PRC1. The biological function of PRC1 was determined by MTT proliferation, colony formation assay, cell cycle, and apoptosis assays. Then, an in vivo tumor formation assay was conducted to explore the effects of PRC1 on tumor growth.

**Results:**

The mRNA and protein expression levels of PRC1 were highly expressed in colon cancer tissues and cell lines. PRC1 expression was associated with clinicopathological characteristics and overall survival of patients with colon cancer. Knockdown of PRC1 could decrease proliferation and colony forming ability of colon cancer cells, as well as arrested more cells at G2/M phase and promoted cell apoptosis. In cancer cells, the expression pattern of protein regulators included in cell cycle and apoptosis progress were reverted by PRC1 down-regulation. Additionally, PRC1 down-regulation could suppress colon tumor growth and differentiation.

**Conclusions:**

We confirmed that PRC1 was overexpressed in colon cancer and was associated with poor prognosis of colon cancer patients. PRC1 down-regulation could arrest cell cycle at G2/M stage, inhibit proliferation, and elicit apoptosis. These findings showed the potential of PRC1 to be used for therapeutic approaches in colon cancer.

## Background

Colon cancer is the third most common cancer, accounting for around 10% of all diagnosed cancers worldwide [1]. Although nationwide screening programs cause an increase in colon cancer incidence, lifestyle [2], heredity [3] and environmental factors [4] might be attributable to the increased the incidence. However, the reasons of this increase are not exactly understood. Patients with colon cancer display a wide range of symptoms, such as abdominal pain, anaemia, disorders of bowel habits, and rectal bleeding. For diagnosis of colon cancer, colonoscopy and computed tomography colonography are increasingly used, with histology as identification [5]. Moreover, researchers have confirmed the potential of identifying patients with colon cancer according to gene expression signatures [6, 7]. Accordingly, gene-based therapy may be useful to eradicate colon cancer, which has been proved as a promising therapeutic strategy [8, 9]. Here, we investigated whether protein regulator of cytokinesis (PRC1) might function as the diagnosis and prognosis of colon and whether showed a potential in targeted treatments of colon cancer.

PRC1 is composed of 620 amino acids, which is structured into Lys/Arg-rich, MT-binding, dimerization, and rod domains [10–12]. PRC1 variably resides in different types of tissues and tightly affects cell division [13]. PRC1 copy number changes and missense mutations disrupt the tight spatiotemporal regulation of cytokinesis [14]. The deregulation of PRC1 contributes to tumorigenesis and cancer process [14, 15]. A recent study confirmed that the dysregulation of PRC1 might be responsible for biochemical recurrence in patients with prostate cancer, implying that PRC1 could be considered as a prognostic indicator [16]. Consistently, a previous report revealed PRC1 was accumulated in the breast cancer, and PRC1 promoter exhibited cancer-specific activity [17]. Besides, evidence suggests that PRC1 is involved in breast tumorigenesis, and it is a promising target for clinical treatment of breast cancer [16]. However, it is still unclear whether PRC1 shows similar expression profile and what roles PRC1 exactly plays in colon cancer.

PRC1 was reported as the second highest ranking gene studied to be correlated with a high grade of chromosomal instability in diverse tumors [18]. Its overexpression is associated with enhanced aneuploidy which leads to worse patient outcome [14, 15, 19]. Genetic abnormalities may cause the deregulation of PRC1 since genetic events such as missense mutations, deletions and amplifications are circularly determined by genomic profiling [20, 21]. Besides, PRC1 is deregulated by signaling pathways at transcriptional level, such as Wnt, p53 and non-estrogen receptor in various cancer, indicating that signaling transduction cascades may be therapeutically utilized to govern rates of cytokinesis defects [22–24]. Consistently, comprehensive analysis of its deregulation is conducive to develop new therapeutic candidates for colon cancer.

Given these confirmations of PRC1 functioning in cancer disease, it is unclear whether PRC1 operates in colon cancer. In this study, we sought to analyze the expression profile of PRC1 in colon cancer as well as the potential affinity between PRC1 and cancer prognosis. Next, we knocked down PRC1 to study its biological function in colon cancer. Our findings demonstrated the involvement of PRC1 in colon cancer, and highlighted its potential as a diagnostic, therapeutic, and prognostic maker for colon cancer.

## Methods

### Patients and tissue samples

A total of 40 patients (20 males and 20 females) ranged 41–85 years (mean, 68.1 years) undergoing surgical resection of colon carcinoma at the Center of Tumor, Inner Mongolia People’s Hospital (Inner Mongolia Autonomous Region, China) between July 2014 and March 2015 were selected for this study. The clinical characteristics of included patients are shown in Additional file 1: Table S1. The colon carcinoma tissues and paired adjacent normal tissues were obtained from surgeries. None of patients received treatment prior to surgery, such as radiotherapy, chemotherapy, biotherapy, or other combination therapies. All samples were confirmed by histopathology as colon carcinoma or adjacent normal colon tissue. Then, tissues were frozen quickly in liquid nitrogen. All patients included in this study had signed written informed consents. This study obtained permission from the Research Ethics Committee of Inner Mongolia People’s Hospital.

### Bioinformatics analysis

The mRNA expressions of PRC1 in colon and normal tissues were obtained from Oncomine (https://www.oncom ine.org/resou rce/login.html) as follows: Sabates-Bellver Colon [25], Hong Colorectal [26], and Skrzypczak Colorectal [27].

### Immunohistochemistry (IHC) and scoring

IHC was performed on a tissue microarray (TMA) slide. The colon cancer TMA (Cat: HCol-Ade180Sur-08) purchased by the Shanghai Outdo Biotech Co., Ltd (Shanghai. China) was chosen for evaluating PRC1 expression. This TMA contained 90 patients with colon cancer who underwent surgical resection between July 2004 and June 2009. The final followed up date was August 2015 and the follow-up time was 5–10 years. The mean age of the patients was 67.7 years (range, 47–90 years), including 45 males and 45 females. Meanwhile, the clinical characteristics of these patients are described in Additional file 1: Table S2. This microarray contained colon cancer tissues and paired normal adjacent tissues from 90 patients. TMA slide was cut into 4-μm-thick section and then were deparaffinized. Antigen retrieval was performed by microwaving in citric acid (pH 6.0) for 5 min, followed by incubation with the primary antibody against PRC1 (51248, Abcam, Cambridge, UK) at 4 °C overnight. After incubation with secondary antibody, images were collected and evaluated blindly by two independent pathologists. The two-way scoring system was used for staining quantification. A semi-quantitative method was resorted to assess the staining intensity with a four point scale: no staining (0), weak staining (1), moderate staining (2), and strong staining (3). Meanwhile, the proportion of positively stained cells was determined as following: 0% (0), 1–25% (1), 26–50% (2), 51–75% (3), and 76–100% (4). The final expression level of protein, ranging from 0 to 12, was calculated by multiplying the intensity score and the proportion score. The average immunohistochemistry score of PRC1 was used as a cut-off to divide the cases into low expression group and high expression group.

### Cell culture

Human colon cancer cells lines (HCT116, SW480, Caco-2, and HT-29) and normal human colon epithelial lines (CCD-18Co) were provided by the Institute of Biochemistry and Cell Biology of the Chinese Academy of Sciences (Shanghai, China). HCT116 cells were cultured in McCOY’s 5A (GIBCO-BRL, Invitrogen, Carlsbad, CA, USA); and CCD-18Co, SW480, Caco-2, and HT-29 cells were cultured in RPMI-1640 (Gibco, Carlsbad, CA, USA). All the culture media were supplemented with 10% fetal bovine serum (FBS). Meanwhile, all cells were cultured in a humidified incubator under standard culture condition (5% CO_2_, 37 °C).

### Short hairpin RNA (shRNA) transfection of colon cancer cell line SW480, HT-29 and HCT116

Two human PRC1 shRNA (shPRC1#1 5′-CCTGAAGGAAAGACTCATCAA-3′ and shPRC1#2 5′-CAGGAACATTCAAAGGCATTT-3′) and control-shRNA were synthesized by GenePharma (Shanghai, China). shPRC1-1 and shPRC1-2 were inserted into pcDNA3.1-PRC1 vectors (Genechem, Shanghai, China) for specifically targeting PRC1 mRNA. For the control lentiviral, shControl sequence (did not target any known gene) was inserted into pcDNA3.1 (Addgene). The construction of the recombinant plasmid was resorted to GenePharma (Shanghai, China). Viruses were kept in the PEG-it virus reagent (System Biosciences, Piscataway, NJ, USA) and maintained at -80 °C. When cells (SW480, HT-29, and HCT116) reached 50–60% confluence, the constructed shRNAs and shControl were transfected into cells using Lipofectamine 2000 (Invitrogen, Life Technologies) according to the manufacturer’s instructions. Successful knockdown of PRC1 expression was tested by quantitative real-time polymerase chain reaction (qRT-PCR) and western blotting.

### RNA extraction and qRT-PCR

Total RNA was extracted from frozen tissues and cultured cells using TRIzol Kit (Invitrogen) based on the manufacturer’s instruction. The complementary DNA (cDNA) was reversely transcribed using the SuperScript III First-Strand Kit (Invitrogen). qRT-PCR reaction was conducted on the ABI7500 Real-Time PCR (Applied Biosystems, Foster City, CA, USA) by using SYBR Premix Ex Taq II (Takara, Dalian, China). GAPDH was used for a reference gene. The relative expression of PRC1 was quantified using 2^−△△CT^ method. All experiments were performed in triplicate. The Primer sequences were as follows (synthesized by Sangon Biotech (Shanghai, China)): PRC1 forward, 5′-TAGACCACACCCCAGACACA-3′ and reverse, 5′-GTGGCCACAGCTTCTCTTTCP-3′; and GAPDH forward, 5′-GCAAATTCCATGGCACCGT-3′, and reverse, 5′-GCCCCACTTGATTTTGGAGG-3′.

### Western blotting assay

The expression levels of PRC1 in cultured cell lines and tumor tissues were detected by western blotting. Total proteins from cells and tissues were extracted and lysed by RIPA buffer (Beyotime, Beijing, China) containing protease and phosphatase inhibitors (Roche, Basel, Switzerland). The concentration of extracted protein was examined using Bio-Rad Protein Assay kit (Bio-Rad, Hercules, CA, USA). Protein extracts (30 μg) were separated on 10–12% sodium dodecyl sulfate–polyacrylamide gel electrophoresis (SDS-PAGE) and then transferred to polyvinylidene fluoride (PVDF) membranes (Millipore, Bedford, MA, USA). Commercial primary antibodies including PRC1 (santa cruz; #8356; 1:1000 dilution), GAPDH (abcam; #ab181602; 1:10000), cyclin B1 (CST; #4138; 1:1000), cyclin dependent kinase 1 (Cdc2; CST; #9116; 1:1000), P21 (Santa cruz; #397; 1:1000), cell division cycle 25c (Cdc25c; CST; #4688; 1:1000), and P27 (CST; #3686; 1:1000) were incubated with the PVDF membrane overnight at 4 °C. The primary antibodies were then probed using the appropriate secondary antibodies at room temperature for 1 h, followed by ECL Western blotting Detection System (Amersham, Piscataway, NJ, USA) for detecting protein signal band. GAPDH served as an endogenous reference and each experiment was run in triplicate. The results of western blotting bands were quantified using Image J software (Rawak Software Inc, Stuttgart, Germany).

### Cell viability assay

The effect of PRC1 on the viability of colon cancer cell lines was determined by 3-[4,5-dimethylthiazol-2-yl]-2,5-diphenyltetrazolium bromide (MTT) (Sigma-Aldrich) assay as described previously [28]. Briefly, shPRC1 (shPRC1-1 and shPRC1-2) and shControl were used to transfect the colon cancer cells (SW480, HT-29, and HCT116). After lentiviral transduction, the cells were seeded at 2.5 × 10^4^ cells/well in 96-well plates, followed by incubation at 37 °C in an atmosphere containing 5% CO_2_ for 7 days. From day 3 to 7, 100 mL of MTT (5 mg/mL) was added and incubated for 4 h each day. Afterwards, the entire supernatant was discarded and then 150 μL dimethyl sulfoxide was added to each well to completely dissolve the formazan. The plates were lightly shaken on the table for 10 min, and optical density values were measured at a wavelength of 490 nm using a spectrophotometer microplate reader (BioTek, Winooski, VT, USA). All analysis was done three times in triplicate.

### Colony formation assay

Transfected SW480, HT-29, and HCT116 cells (1000 cells/well) were seeded into 6-well plates with fresh medium containing 10% FBS. Cells were cultured for 14 days, and the medium was changed every 4 days. Subsequently, the cells were fixed with methanol for 15 min and stained with 1% crystal violet (Solarbio) for 20 min at room temperature. The cells were dried and images were captured using a microscope (Olympus, FV500-IX71, Japan) to count the number of colonies containing > 50 cells.

### Cell cycle analysis

Cell cycle analysis was conducted to verify whether PRC1 regulated the cell cycle. In short, the SW480 and HCT116 cells were transfected with shPRC1 (shPRC1-1 and shPRC1-2) and shControl for 5 days. Then, transfected cells were harvested and fixed with 70% ice-cold ethanol overnight at 4 °C. Cell suspensions were stained with 50 μg/mL of propidium iodide (PI, containing 100 μg/mL RNAase) and analyzed using a flow cytometry (BD Biosciences, San Diego, CA, USA). The experimental procedures were carried out three times in triplicates.

### Apoptosis assay

Flow cytometry was used to detect the apoptotic rate. Colon cells (SW480 and HCT116) transfected with shPRC1 (shPRC1-1 and shPRC1-2) or shControl were plated into 6-well plates and grown to 90% confluence. Following incubation, cell suspensions were harvested and incubated with 10 μL Annexin V-FITC (Beijing Biosea Biotechnology co., LTD., Beijing, China) staining for 10–15 min at room temperature avoiding lights. Then, the cells were analyzed using a flow cytometer (BD Biosciences, San Diego, CA, USA).

### Mice experiments

Four-week old male athymic BALB/c nude mice were provided by SLAC Laboratory Animal Co. (Shanghai, China). The experimental protocols were permitted by the Institutional Animal Care and Use Committee of Jinling Hospital. Mice were kept in a specific pathogen-free environment and randomly divided into two groups (7 mice per group). Xenograft tumor models were established by subcutaneously injecting of stable shControl- or shPRC1-1-expressing HCT116 cells (3 × 10^7^) into the left flank of nude mice. The volume and weight of the tumors were measured every three days for a total of three weeks. Tumor volume (V, mm^3^) was measured using calipers and calculated according to the formula: V = length × width^2^ × 0.5. After 21 days, mice were euthanized and tumors were extracted for further analysis. These tissues were fixed in 4% paraformaldehyde for 24 h. The tissues were processed for hematoxylin and eosin (H&E) staining as well as immunohistochemical staining for Cdc2, Cyclin B1, ki67, and PRC1. The primary antibody used in this analysis was as follows: PRC1 (abcam; #51248: 1:500), Cyclin B1 (CST; #4138; 1:1000), Cdc2 (CST; #9116; 1:1000), and Ki67 (CST; #9449; 1:400). The images were obtained under a brightfield microscopy (Olympus, Tokyo, Japan).

### Statistical analysis

All data were analyzed using GraphPad Prism 6 software (GraphPad, San Diego, CA, USA) and shown as the mean ± standard deviation (SD). Differences between two groups were analyzed using the *t*-test, and comparisons among multiple groups were applied by one-way analysis of variance (ANOVA). Chi-square test was used to assess the association between PRC1 level and clinical characteristics of colon cancer patients. The cut-off value to divide patients into high and low expression groups was defined as the average expression of PRC1. Kaplan–Meier method was used to plot survival curves and log-rank test was utilized to compare difference. Cox proportional hazards model was chosen to evaluate independent prognostic factor. *P* < 0.05 was considered statistically significant.

## Results

### PRC1 was high expression in colon cancer tissues and colon cell lines

**Fig. 1:**
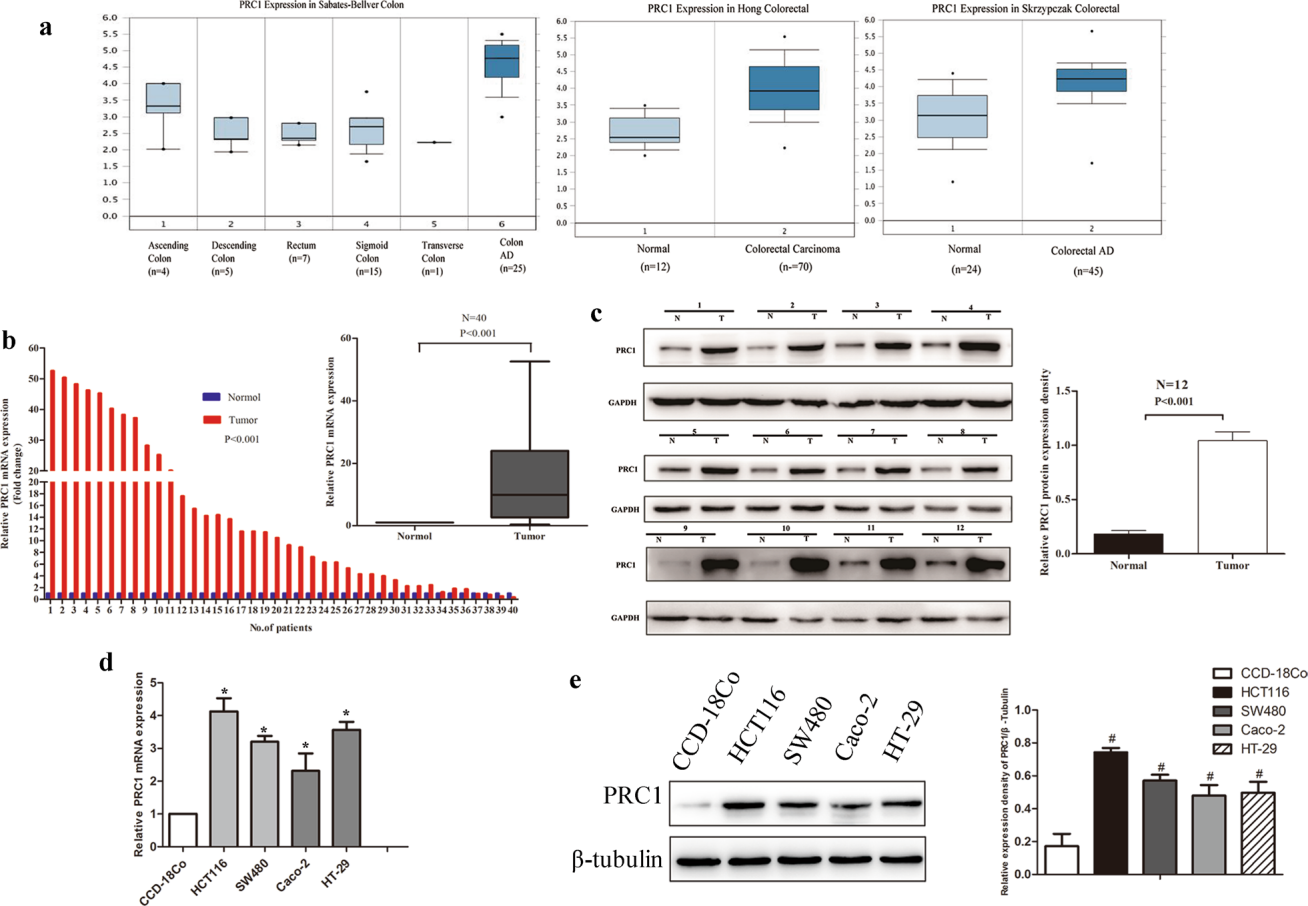
PRC1 was overexpressed in colon cancer tissues and cells. **a** Oncomine data showed that PRC1was highly expressed in colon tissues compared with normal tissues. **b** The mRNA expression level of PRC1 was significantly up-regulated in colon cancer tissues compared with adjacent normal tissues in 40 paired tissues. **c** The PRC1 protein expression level was significantly elevated in colon cancer tissues compared with adjacent normal tissues in selected 12 paired tissues. **d**, **e** The expression level of PRC1 was higher in the colon cancer cell lines (HCT116, SW480, Caco-2, and HT-29) than in the normal human epithelial line (CCD-18Co) on mRNA (**d**) and protein level (**e**). Data were presented as the mean ± standard deviation (SD). ^#^*P* < 0.001 and *P < 0.05

First, we investigated the PRC1 expression in datasets available from Oncomine (https://www.oncomine.org). The data of Sabates-Bellver colon, Hong colorectal, and Skrzypczak colorectal showed that PRC1 was overexpressed in colon cancer compared to normal tissues (Fig. 1a). Then, PRC1 expression level was examined in 40 colon cancer tissues and paired adjacent normal tissues by qRT-PCR. The results indicated that PRC1 expression was significantly higher in cancer tissues than that in normal tissues (*P* < 0.001, Fig. 1b). Moreover, we selected 12 pairs of colon tumor tissues and adjacent normal tissues to detect the expression of PRC1 protein. Compared with the normal tissues, the PRC1 protein expression level was significantly elevated in colon cancer tissues (*P* < 0.001, Fig. 1c). Simultaneously, we examined PRC1 in cell lines by qRT-PCR and western blotting. The PRC1 level was obviously upregulated in colon cell lines (HCT116, SW480, Caco-2, and HT-29) compared with the human colon epithelial line CCD-18Co (*P* < 0.05, Fig. 1d, e).

### PRC1 was associated with clinicopathlogical parameters and poor prognosis in colon cancer

**Fig. 2:**
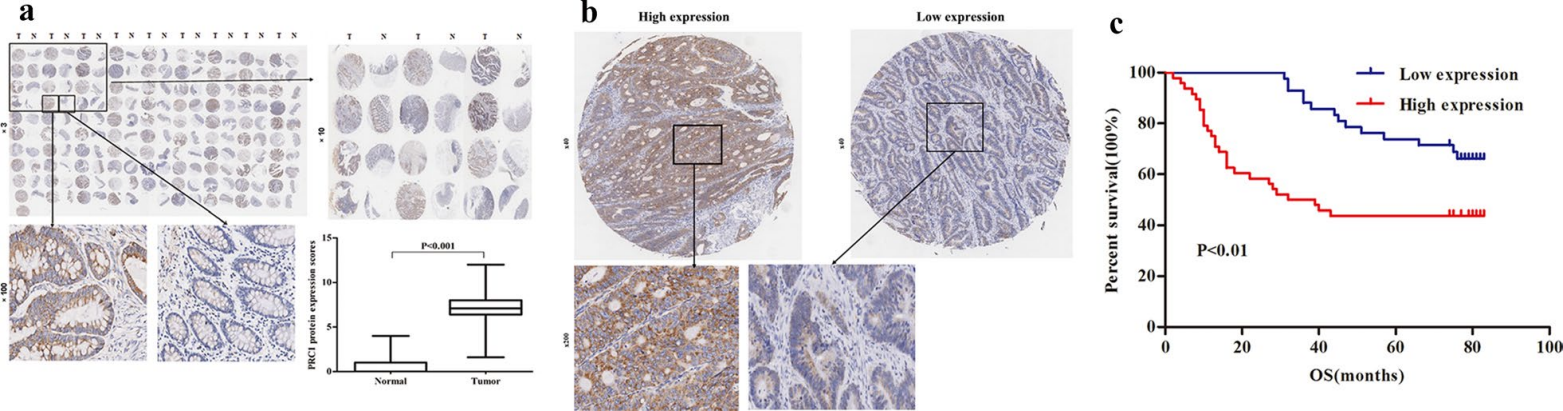
PRC1 was associated with poor prognosis in colon cancer. **a** IHC images of PRC1 protein in 90 colon cancer tissues and adjacent normal tissues obtained from TMA. The PRC1 protein expression score in tumor tissues was significantly higher than normal tissues. **b** Representative images of PRC1 IHC staining from colon cancer TMA. Left: high PRC1 expression in colon cancer (≥ 6.2), right: low PRC1 expression in colon cancer (< 6.2). **c** OS was poor in patients with high expression of PRC1 than in those with low expression among 90 colon cancer patients. *IHC* immunohistochemistry, *TMA* tissue microarray, *OS* overall survival

In order to further explore the expression pattern of PRC1 in colon cancer, we conducted IHC in an established TMA, which composed of 90 paired colon cancer tissues and neighboring non-cancerous tissues. As shown in Fig. 2a, results from TMA suggested that the protein level of PRC1 was universally higher expressed in colon cancer tissues relative to the adjacent normal tissues (*P* < 0.001). In addition, the 90 patients could be grouped into low-expression and high-expression groups according to the immunohistochemical score of PRC1, using 6.2 as a cut-off value (Fig. 2b). The relationship between PRC1 expression and clinicopathlogical parameters of patients with colon cancer is shown in Table 1. The results demonstrated that high expression of PRC1 was associated with high TNM stage (*P* < 0.01), large tumor size (> 5 cm, *P* < 0.05), and lymph node metastasis (*P* < 0.05); while there was no relationship between PRC1 expression and gender (*P* = 0.673) as well as age (*P* = 0.539).

**Table 1:** Correlation between PRC1 protein expression and clinicopathological parameters of colon cancer patients (n = 90)

Characteristics	Number	PRC1 protein expression		
		Low < 6.2	High ≥ 6.2	P-value
All patients	90	42	48	
Gender				
Male	45	20	25	0.673
Female	45	22	23	
Age (years)				
< 65	33	14	19	0.539
≥ 65	57	28	29	
Size of tumor				
< 5 cm	49	28	21	0.029*
≥ 5 cm	41	14	27	
Lymph nodes				
0 nodes	55	31	24	0.021*
1–3 nodes	35	11	24	
TNM stages				
I (Ia, Ib)	47	29	18	0.003*
II–IV	43	13	30	

* *P* < 0.05. Chi-square test

Kaplan–Meier survival analysis revealed that colon cancer patients with high expression level of PRC1 had a poorer overall survival (OS, Fig. 2c). Additionally, cox regression analysis confirmed that PRC1 was an independent prognostic factor for colon cancer (95% CI: 1.840–12.346, *P* < 0.001). Moreover, results showed that lymph node metastasis, p-TNM stages and PRC1 expression were significantly associated with OS (all *P* < 0.05, Table 2).

**Table 2:** Cox regression analysis of PRC1 protein expression and other clinical prognostic factors for overall survival in colon cancer patients (n = 90)

Factors	HR	Univariate analysis 95% CI	P	HR	Multivariate analysis 95% CI	P
Gender (female/male)	1.439	0.776–2.667	0.248	1.710	0.914–3.201	0.093
Age (≥ 65/ < 65 years)	1.561	0.796–3.060	0.195	1.705	0.861–3.374	0.126
Size of tumor (> 5 cm/ ≤ 5 cm)	2.085	1.112–3.908	0.022*	1.840	0.934–3.626	0.078
Lymph node metastasis (N1–3/N0)	2.550	1.377–4.724	0.003*	2.697	1.432–5.078	0.002*
p-TNM stages (II + III + IV/I)	2.950	1.501–5.800	0.002*	2.393	1.196–4.788	0.014*
PRC1 expression (high/low)	5.853	2.285–14.997	0.000*	4.766	1.840–12.346	0.001*

*HR* hazard ration, *95% CI* 95% confidence interval

* P < 0.05

### Silencing of PRC1 inhibited cell viability and colony forming ability of colon cancer cells

**Fig. 3:**
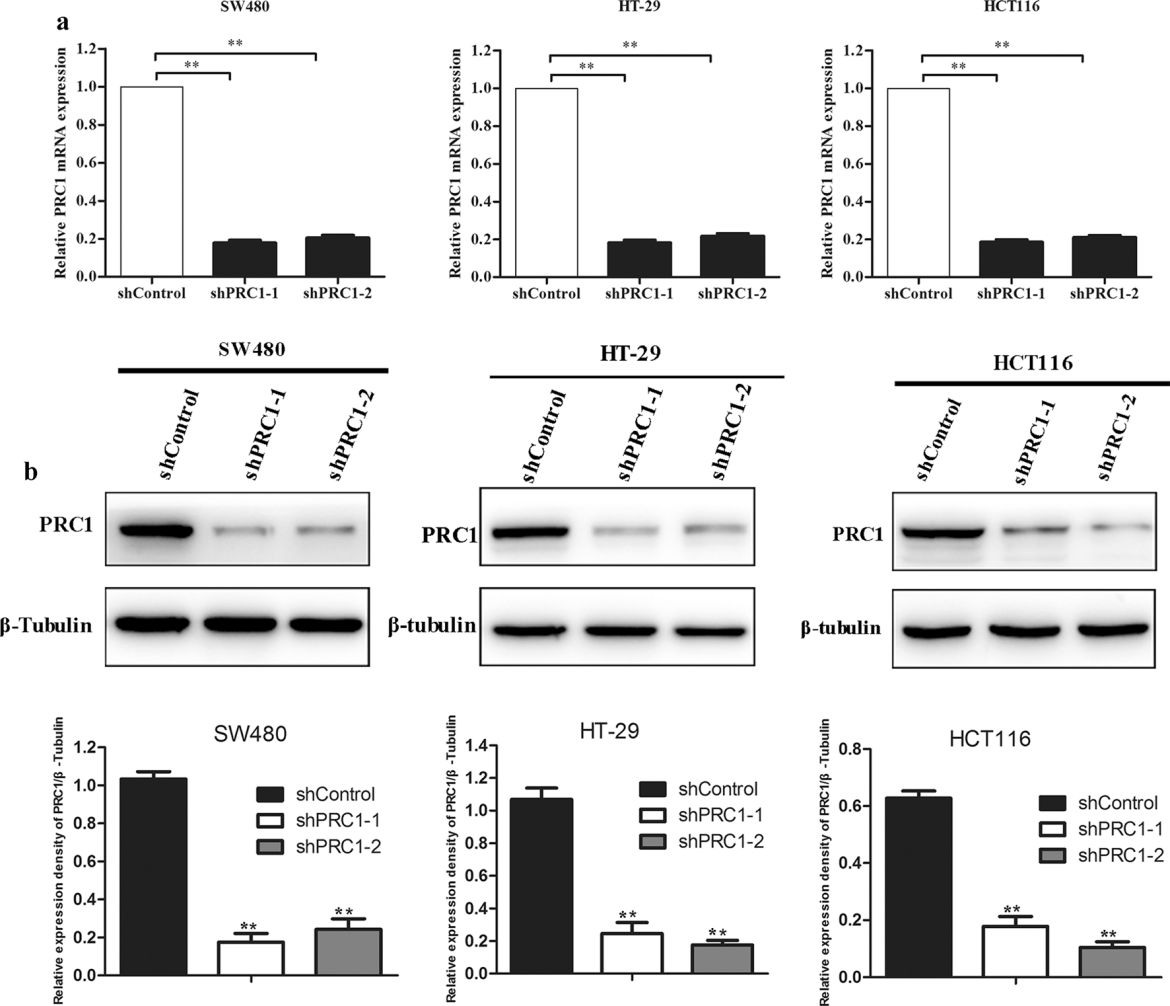
The interfering efficiency of two shRNA targeting PRC1 (shPRC1-1 and shPRC1-2) was exaimed by qRT-PCR (**a**) and western blotting (**b**). Compared with normal group (shControl), the expression levels of PRC1 in shPRC1 transfected colon cancer cell lines (SW480, HT-29, and HCT116) were significantly decreased. **P < 0.01

**Fig. 4:**
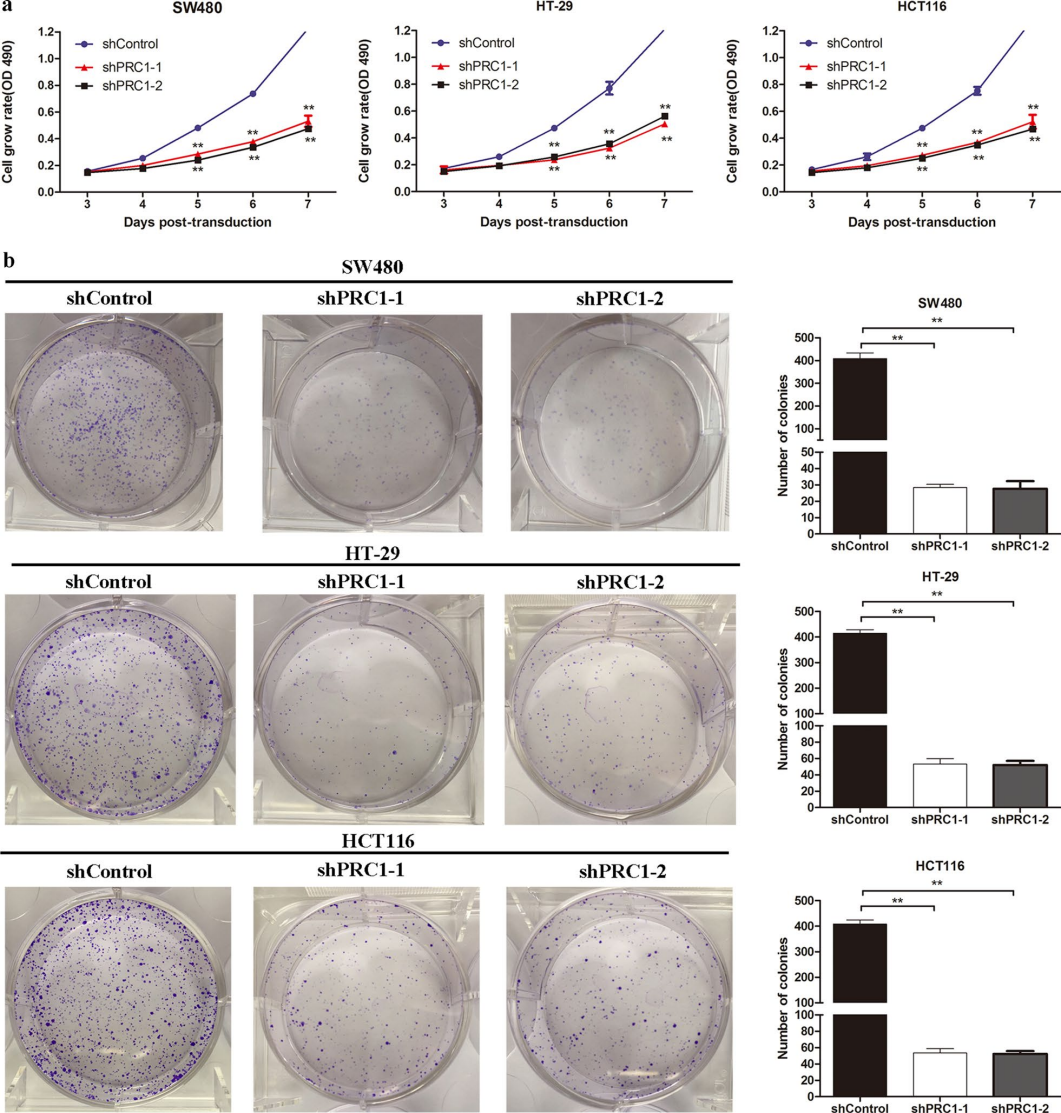
Silencing of PRC1 inhibited cell viability and colony forming action ability of colon cancer cells. **a** The cell grow rate of colon cancer cells (SW480, HT-29, and HCT116) was decreased in shPRC1 transfected groups (shPRC1-1 and shPRC1-2). **b** PRC1 knockdown inhibited SW480, HT-29, and HCT116 cells proliferation. **P < 0.01

To explore the biological function of PRC1 in colon cancer cells, we established the colon cancer cells (SW480, HT-29, and HCT116) with lentivirus mediated PRC1 knockdown, which was validated by qRT-PCR and western blotting. The results confirmed that both mRNA and protein expression levels of PRC1 were significantly suppressed in sh-PRC1 transfected cell lines (*P* < 0.05) (Fig. 3a, b). MTT and colony forming assays were performed to determine the influence of PRC1 on cell proliferation. As shown in Fig. 4a, cell proliferation was significantly inhibited in the shPRC1 groups (shPRC1-1 and shPRC1-2) compared with that in shControl group in SW480, HT-29, and HCT116 cells (*P* < 0.01). In addition, colony formation assay was conducted to evaluate the colony formation ability of colon cells. As presented in Fig. 4b, the decreased number of colonies was observed in the cells transfected with shPRC1 (shPRC1-1 and shPRC1-2) (*P* < 0.01).

### Silencing of PRC1 arrested more cells in G2/M phase and promoted apoptosis of colon cancer cells

**Fig. 5:**
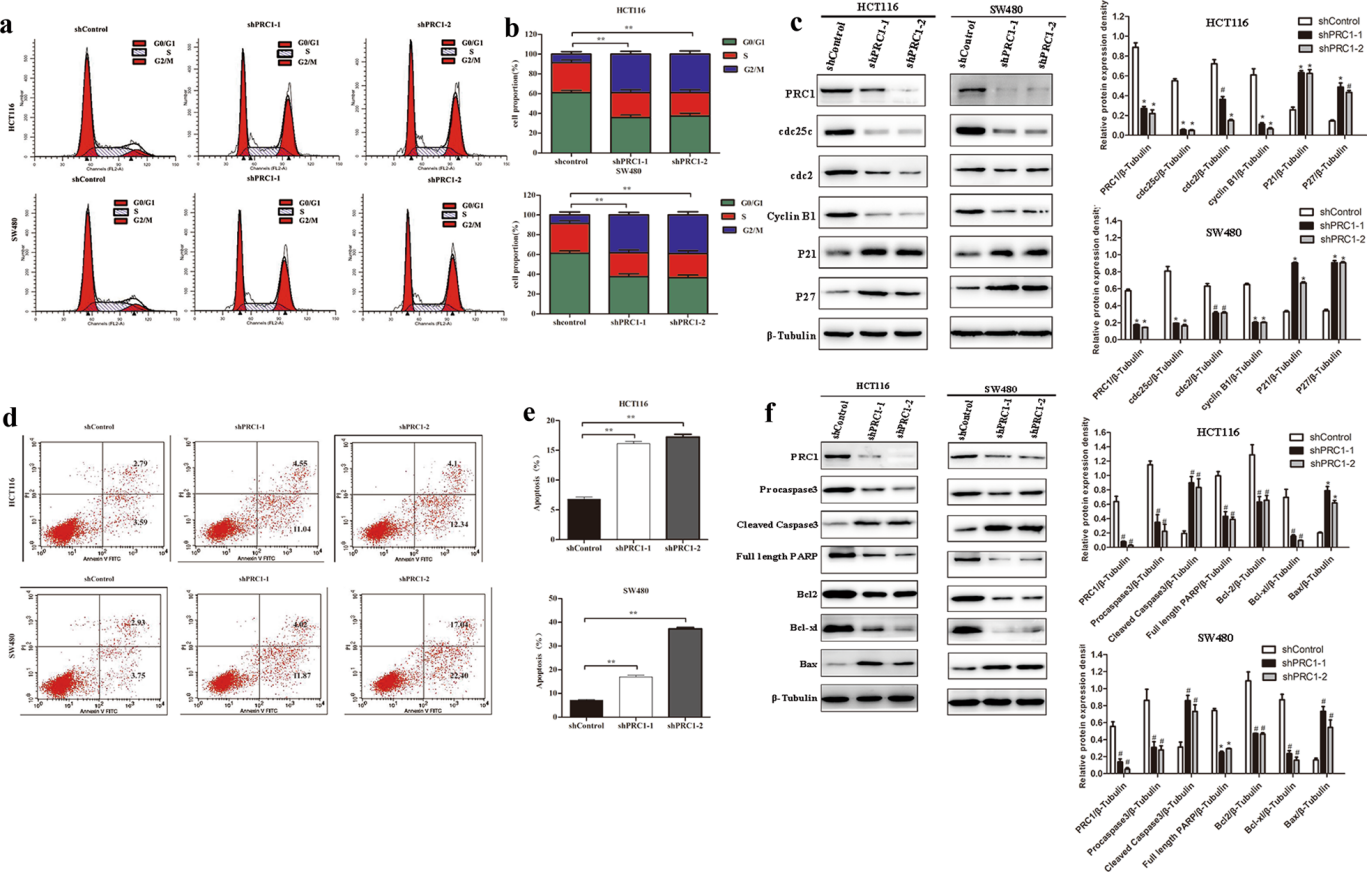
Silencing of PRC1 arrested more cells in G2/M phase and induces cell apoptosis in HCT116 and SW480 cells. **a** Cell cycle distribution of HCT116 and SW480 cells in the three groups (shControl, shPRC1-1, and shPRC1-2). **b** Percentage of cells in cell cycle phase G0/GA, S, and G2/M. **c** The expression levels of cell cycle related proteins (Cdc25c, Cdc2, Cyclin B1, P21, and P27) were detected by western blotting. **d** Cell apoptosis in HCT116 and SW480 cells detected by flow cytometry. **e** Apoptosis rate in HCT116 and SW480 cells in the three groups. **f** The expression level apoptosis related proteins (pro caspase 3, cleaved caspase 3, full length PARP, Bcl2, Bcl-xl, and Bax) was detected by western blotting. Data were expressed as the mean ± standard deviation (mean ± SD). *P < 0.05, **P < 0.01, # *P* < 0.001

Cell cycle distribution of SW480 and HCT116 cells in each group (shControl, shPRC1-1, and shPRC1-2) was evaluated by PI staining. As shown in Fig. 5a, b, silencing of PRC1 expression in HCT116 and SW480 cells, compared with the shControl group, led to a significant increase in G2/M phase arrest and a decrease in the G0/G1 cell population (*P* < 0.01). To further elucidate molecular mechanisms of PRC1 regulating cell proliferation, we detected the cell cycle-related proteins levels in HCT116 and SW480 cells by western blotting. Compared with shControl group, cell cycle inhibitors at G1 phase (p21 and p27) were significantly up-regulated (*P* < 0.05), while cell cycle regulatory proteins (Cdc25c, Cdc2, and Cyclin B1) were down-regulated in the cells transfected with shPRC1-1 and shPRC1-2 (*P* < 0.05, Fig. 5c). Moreover, flow cytometry was performed for detection of cell apoptosis of HCT116 and SW480 cells. Compared with the shControl group, the apoptosis rate was increased in the shPRC1 transfected cells (*P* < 0.01) (Fig. 5d, e). Furthermore, the expression levels of apoptosis-related proteins were detected by western blotting. Our results showed that PRC1 positively regulated cleaved caspase 3 and Bax, as well as negatively regulated full length PARP, Bcl2 and Bcl-xl (*P* < 0.05) (Fig. 5f). These results provided the evidences that PRC1 showed oncogenic effects via regulating G2/M cell cycle and cell apoptosis.

### Silencing of PRC1 mitigated the growth of tumors

**Fig. 6:**
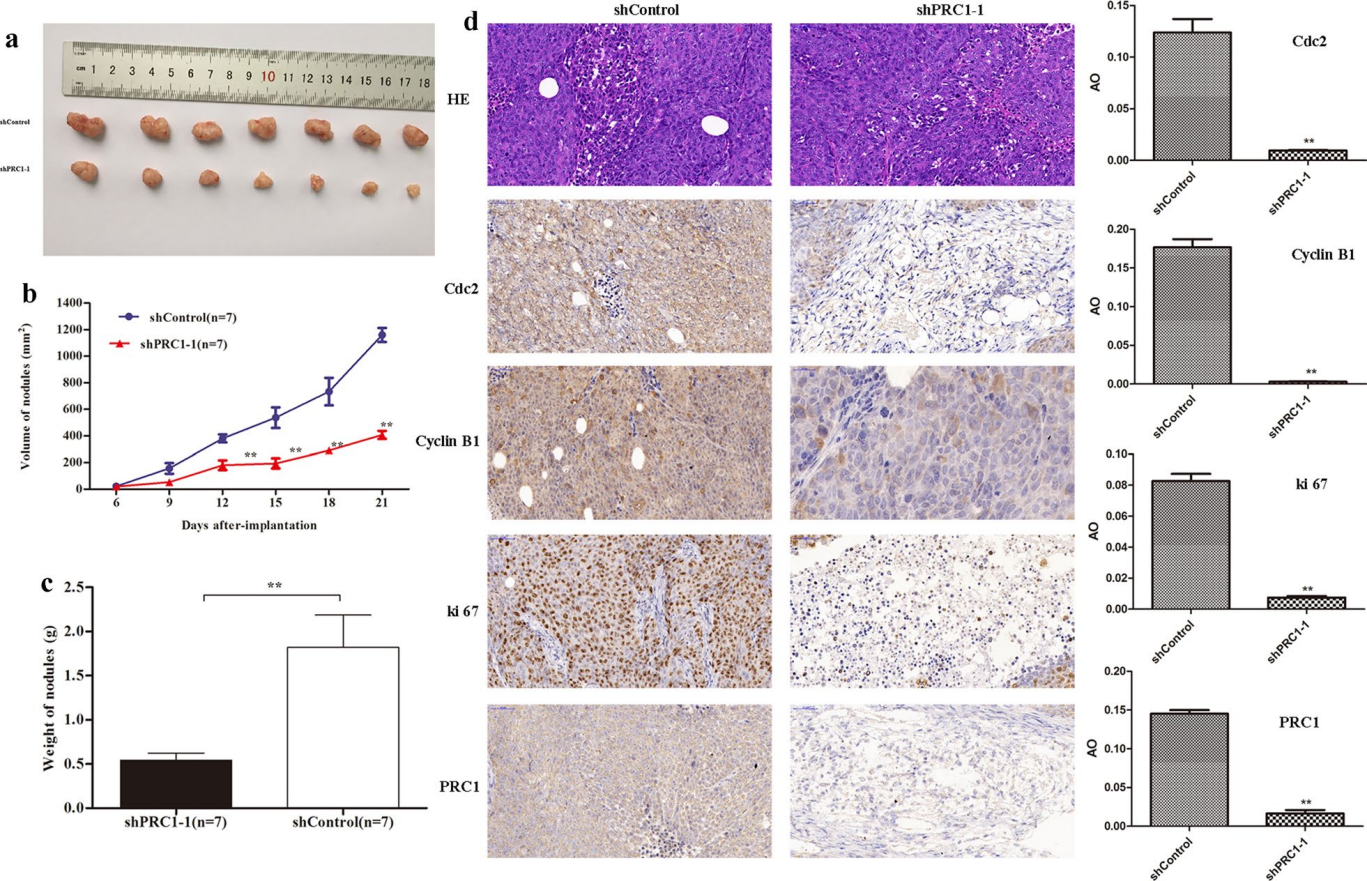
Knockdown of PRC1 inhibited tumor growth in vivo. **a** Xenograft tumor formation of HCT116 cells transfected shControl and shPRC1-1. **b**, **c** The volume and weight of tumor were calculated. **d** The resected tumors were subjected to H&E staining and IHC staining for Cdc2, cyclin B1, Ki67 and PRC1 (× 200 magnification). *H&E* hematoxylin–eosin, *IHC* immunohistochemical, *AO* average optical. Data were expressed as the mean ± standard deviation (mean ± SD). **P < 0.01

Finally, we established xenograft tumor model to investigate the effect of PRC1 on tumor growth in vivo. Results suggested that the tumors of the PRC1 knockdown group (shPRC1-1) were significantly smaller and lighter than those of the control group (*P* < 0.01) (Fig. 6a–c). Subsequently, immunohistochemical staining of xenograft tissues collected from nude mice demonstrated that knockdown of PRC1 could significantly inhibit the expression of cancer differentiation-related proteins (Cdc2, Cyclin B1, and ki67) (*P* < 0.05, Fig. 6d). Overall, these findings illustrated that inhibition of PRC1 could suppress colon tumor growth and differentiation.

## Discussion

In this study, we observed that PRC1 was significantly up-regulated in colon tissues and cell lines. The expression level of PRC1 was associated with several clinicophathological characteristics in colon cancer patients, including tumor size, lymph node metastasis, and p-TNM stages; in addition, patients with high expression of PRC1 suggested unfavorable prognosis, which indicated that PRC1 might serve an important role in the development and progression of colon cancer. Furthermore, the biological function of PRC1 was investigated in vitro and in vivo, and results showed that knockdown of PRC1 inhibited proliferation and promoted apoptosis of colon cancer cells. Meanwhile, silencing of PRC1 resulted in more cells arrested at G2/M phase. The results of mice experiment demonstrated that knockdown of PRC1 could inhibit tumor growth. Our data provided more evidence that PRC1 might serve a therapeutic target for colon cancer.

PRC1 is involved in the completion of cytokinesis and is abnormally regulated in a tumor-specific manner. Numbers studies have reported the role of PRC1 in cancer development. Luo et al. found that PRC1 expression levels were significantly higher in prostate cancer (PCa) tissues than those in normal tissues; in addition, overexpression of PRC1 was significantly associated with advanced clinicopathologic features and a shorter biochemical recurrence-free survival in patients with PCa [29]. Bu et al. observed that the mRNA and protein expression levels were up-regulated in high-grade serous ovarian carcinoma (HGSOC) tissues, and knockdown of PRC1 could reduce the proliferation, metastasis, and in vitro multidrug resistance of ovarian cancer cell [30]. Consistent with previous studies, our experiments also indicated that PRC1 was obviously higher in tumor tissues compared with normal paracancer tissues. Meanwhile, its expression level was associated with the clinical indicators and OS of patients with colon cancer, revealing that PRC1 had carcinogenic effect and might be an ideal target for the therapy of colon cancer.

Most notably, our findings showed that silencing of PRC1 could significantly inhibit the proliferation and attenuate the colony forming ability of colon cancer cells. Proliferation is an important feature of life activities and its manifestation at the cellular level is cell division [31]. The accurate entry of cells into the growth and division cycle is a prerequisite for maintaining normal cell proliferation and genome stability. However, the tetraploid and chromosomal instability caused by the failure of accurate cell division can promote the occurrence and development of tumors [32]. Li et al. suggested that PRC1 was essential for cytokinesis and normal cell cleavage, and deregulation of PRC1 protein could cause cell division defects, thereby promoting chromosomal instability, leading to tumor heterogeneity and tumor evolution [33]. Thus, we speculated that abnormal expression of PRC1 led to erroneous cell division, which in turn promoted the development of colon cancer. Subsequently, we explored the effect of PRC1 on the cell cycle and apoptosis of colon cancer cells.

Our results showed that silencing of PRC1 expression arrested cell cycle progress at the G2/M interphase. Meanwhile, the expression levels of cell cycle related proteins were detected by western blotting. Silencing of PRC1 leaded to decrease in the protein expression level of Cdc25c, Cdc2, and Cyclin B1. Previous study revealed that Cdc25c-mediated de-phosphorylation of apoptosis signal-regulating kinase 1 (ASK1) and Cdc25 overexpression blocked ASK1-mediated apoptosis in a cell cycle-dependent manner, showing that Cdc25c functioned in G2/M checkpoint-mediated apoptosis [34]. Furthermore, cyclin B1 interacting through its P-box with Cdc25 activated Cdc25c and subsequently activated Cdc2 kinase [35]. Meanwhile, we noticed that inhibition of PRC1 resulted in increases of P21 and P27. As reported, P21 and P27, possessing the overlapping functions with each other, were involved in the cell cycle exit at G1-phase [36]. What’s more, down-regulation of PRC1 was responsible for the cleavage of caspase 3 and PARP, increased the Bax expression, as well as decreased the Bcl2 and Bcl-xl expression, which led to cell apoptosis [37]. It was therefore probable that PRC1 decrease arrested cell cycle at the G2/M interphase and caused apoptosis through affecting regulator expression. However, how PRC1 affects their expression remains to be determined.

Finally, our findings from xenograft tumor growth assays demonstrated that silencing of PRC1 expression inhibited the weight and volume of the tumor. This inhibition of PRC1 expression resulted in decreased the protein expression levels of Cdc2 and Cyclin B1 in xenograft tumors, which was in consistence with the results from in vitro experiments. Moreover, another importance observation in our studies was that PRC1 down-regulation decreased Ki67 expression. Of note, Ki67 is exclusively expressed within the cell nucleus during interphase while relocates on the chromosomal surface in mitosis, being strikingly associated with proliferative activities [38, 39]. Our findings strongly suggested that PRC1 down-regulation inhibited xenograft-tumor growth in vivo.

To the best of our knowledge, this is the first study to explore the biological function of PRC1 in the pathogenesis of colon cancer. Nevertheless, our study has several limitations. Due to the incomplete follow-up data for 5–10 years, the association between PRC1 expression and clinical features (such as OS, gender, age, size tumor, and so on) has not been investigated in the collected 40 patients. Moreover, the precise pathway through which PRC1 influences colon cancer progression has not been explored. In the subsequent research, we will further reveal the potential regulation pathways for PRC1 in colon cancer.

## Conclusions

The present study depicted the correlation of colon cancer progress to PRC1 overexpression. Our data presented in this study showed that PRC1 was overexpressed in colon cancer and was associated with poor OS. PRC1 knockdown could arrest cell cycle at G2/M stage, inhibit proliferation, and promote apoptosis. These results indicated the potential of PRC1 to be used for therapeutic approaches in colon cancer.

## Supplementary information

**Additional file 1: Table S1.** The clinical characteristics of included patients (n = 40). **Table S2.** Colon cancer clinical and pathological data of patients from tissue microarray (n = 90).

## Data Availability

All data generated or analyzed during this study are included in this published article.

## Abbreviations

PRC1: Protein regulator of cytokinesis 1
PI: Propidium iodide
PVDF: Polyvinylidene fluoride
SD: Standard deviation
ASK1: Apoptosis signal-regulating kinase 1
