# Supplementary material for: Overexpression of protein regulator of cytokinesis 1 facilitates tumor growth and indicates unfavorable prognosis of patients with colon cancer
Source: Cancer Cell Int. 2020 Oct 31;20:528. doi: 10.1186/s12935-020-01618-9 (PMC7603724; doi:10.1186/s12935-020-01618-9)
Supplement: Supplementary file 1 — Additional file 1: Table S1. The clinical characteristics of included patients (n = 40). Table S2. Colon cancer clinical and pathological data of patients from tissue microarray (n = 90). [file 12935_2020_1618_MOESM1_ESM.doc]

**Table S1. The clinical characteristics of included patients (n = 40)**

| **Code** | **Age** | **Gender** | **Tumor size** | **Pathological type** | **Total number of lymph nodes** | **Number of** **positive lymph nodes** | **BMI（Kg/m^2^）** |
| --- | --- | --- | --- | --- | --- | --- | --- |
| Col01 | 73 | Male | 5.5×4×1.5cm | Adenocarcinoma | 15 | 0 | 26 |
| Col2 | 65 | Male | 3.4×2.7×1cm | Adenocarcinoma | 17 | 0 | 23 |
| Col3 | 64 | Female | 5.5×4×2cm | Tubular adenocarcinoma | 12 | 0 | 20 |
| Col4 | 80 | Female | 10×7×1.4cm | Adenocarcinoma | 18 | 6 | 28 |
| Col5 | 64 | Male | 3.3×2.7×1cm | Adenocarcinoma | 14 | 0 | 30 |
| Col6 | 65 | Male | 3.4×2.7×1cm | Adenocarcinoma | 13 | 1 | 26 |
| Col7 | 64 | Female | 5.5×4×2cm | Tubular adenocarcinoma | 22 | 2 | 24 |
| Col8 | 78 | Male | 4×3×1.5cm | Tubular adenocarcinoma | 21 | 0 | 26 |
| Col9 | 77 | Male | 4×4×1.5cm | Tubular adenocarcinoma | 19 | 0 | 22 |
| Col10 | 65 | Male | 4.5×3.5×1.5cm | Tubular adenocarcinoma | 17 | 1 | 21 |
| Col11 | 53 | Male | 3×2.5×1cm | Tubular adenocarcinoma, partial mucinous adenocarcinoma | 16 | 0 | 18 |
| Col12 | 56 | Male | 3×3×0.8cm | Tubular adenocarcinoma | 15 | 0 | 17 |
| Col13 | 76 | Female | 3×3×1cm | Tubular adenocarcinoma | 15 | 1 | 26 |
| Col14 | 77 | Female | 4.5×3×1cm | Tubular adenocarcinoma | 14 | 0 | 25 |
| Col15 | 74 | Male | 5.5×5×1.5cm | Adenocarcinoma | 16 | 0 | 22 |
| Col16 | 74 | Male | 10×4×1.5cm | Mucinous adenocarcinoma | 15 | 4 | 21 |
| Col17 | 80 | Male | 4.5×4×1.5cm | Adenocarcinoma | 14 | 0 | 20 |
| Col18 | 65 | Male | 4×5×1cm | Adenocarcinoma, partial mucinous adenocarcinoma | 12 | 0 | 21 |
| Col19 | 66 | Female | 7×4.5×1.5cm | Adenocarcinoma | 18 | 1 | 22 |
| Col20 | 58 | Female | 3×2×1cm | Adenocarcinoma | 18 | 0 | 23 |
| Col22 | 78 | Female | 5×4×1cm | Tubular adenocarcinoma | 19 | 0 | 24 |
| Col23 | 66 | Male | 4×4×1cm | Tubular adenocarcinoma | 19 | 0 | 23 |
| Col24 | 60 | Male | 7×5.5×4cm | Tubular adenocarcinoma | 20 | 3 | 19 |
| Col26 | 65 | Female | 5.5×4.5×1.5cm | Adenocarcinoma | 22 | 0 | 19 |
| Col27 | 56 | Male | 4×3×1cm | Tubular adenocarcinoma | 21 | 0 | 25 |
| Col28 | 63 | Female | 4×5×1cm | Adenocarcinoma | 25 | 0 | 26 |
| Col25 | 41 | Female | 5×4×1cm | Tubular adenocarcinoma | 24 | 3 | 25 |
| Col27 | 76 | Male | 5.5×4.5×1.5cm | Tubular adenocarcinoma | 23 | 0 | 23 |
| Col28 | 82 | Female | 5.5×3×1cm | Tubular adenocarcinoma | 22 | 0 | 24 |
| Col29 | 55 | Female | 7×5×2cm | Tubular adenocarcinoma | 15 | 5 | 25 |
| Col30 | 70 | Female | 5×4×1.5cm | Tubular adenocarcinoma | 16 | 1 | 26 |
| Col31 | 79 | Male | 5.5×4×1.5cm | Adenocarcinoma | 18 | 0 | 27 |
| Col33 | 65 | Male | 4×3×3cm | Mucinous adenocarcinoma | 12 | 0 | 23 |
| Col34 | 78 | Female | 4.5×3.5×1cm | Tubular adenocarcinoma | 14 | 0 | 22 |
| Col35 | 55 | Female | 2×1.5×1cm | Adenocarcinoma | 16 | 4 | 24 |
| Col36 | 75 | Female | 3×2.5×1.8cm | Tubular adenocarcinoma | 15 | 0 | 23 |
| Col37 | 76 | Male | 4×3.5×2.5cm | Tubular adenocarcinoma | 13 | 5 | 20 |
| Col38 | 60 | Female | 4×2×1.2cm | Tubular adenocarcinoma, partial mucinous adenocarcinoma | 19 | 0 | 21 |
| Col39 | 85 | Female | 3×3×1cm | Tubular adenocarcinoma | 22 | 0 | 22 |
| Col40 | 65 | Female | 4.5×3×1cm | Tubular adenocarcinoma | 24 | 0 | 21 |

**Table S2. Colon Cancer Clinical and Pathological Data of patients from Tissue microarray (n = 90)**

| **Code** | **Age** | **Gender** | **Size of tumor** | **Pathological type** | **Survival status** | **Lifetime**  **/month** |
| --- | --- | --- | --- | --- | --- | --- |
| RDgCol0706A0615 | 78 | Female | 6×5×1.5cm | Adenocarcinoma | Death | 32 |
| RDgCol0706A0616 | 74 | Female | 10×5×1.5cm | Mucinous adenocarcinoma | Death | 7 |
| RDgCol0706A0617 | 81 | Female | 4.5×4×1.5cm | Adenocarcinoma | Death | 14 |
| RDgCol0706A0618 | 64 | Female | 5×5×1cm | Adenocarcinoma | Survival | 83 |
| RDgCol0706A0619 | 62 | Male | 7×6×1.5cm | Adenocarcinoma | Survival | 83 |
| RDgCol0706A0620 | 57 | Male | 3×2×1cm | Adenocarcinoma | Death | 10 |
| RDgCol0706A0622 | 76 | Male | 5×4×1cm | Tubular adenocarcinoma | Survival | 83 |
| RDgCol0706A0623 | 60 | Female | 5×4×1cm | Tubular adenocarcinoma | Survival | 83 |
| RDgCol0706A0624 | 60 | Female | 7×6×4cm | Tubular adenocarcinoma | Death | 40 |
| RDgCol0706A0626 | 61 | Male | 6×4.5×1.5cm | Adenocarcinoma | Survival | 83 |
| RDgCol0706A0627 | 54 | Female | 4×3×1cm | Tubular adenocarcinoma | Death | 9 |
| RDgCol0706A0628 | 53 | Male | 5×5×1cm | Adenocarcinoma | Survival | 83 |
| RDgCol0709A0640 | 74 | Male | 5×4×1cm | Tubular adenocarcinoma | Survival | 83 |
| RDgCol0709A0641 | 74 | Male | 4.5×3×1.5cm | Tubular adenocarcinoma | Death | 47 |
| RDgCol0709A0642 | 70 | Female | 9×6×1cm | Mucinous adenocarcinoma | Survival | 82 |
| RDgCol0709A0645 | 76 | Female | 4×2×0.6cm | Adenocarcinoma | Survival | 82 |
| RDgCol0709A0646 | 57 | Male | 6×3×1cm | Adenocarcinoma | Death | 43 |
| RDgCol0709A0647 | 66 | Female | 7×5×2cm | Adenocarcinoma | Death | 76 |
| RDgCol0709A0648 | 65 | Male | 9×6×1.5cm | Tubular adenocarcinoma | Survival | 82 |
| RDgCol0709A0649 | 50 | Female | 2.5×2×1cm | Tubular adenocarcinoma | Death | 27 |
| RDgCol0709A0652 | 80 | Male | 4.5×4×1.5cm | Adenocarcinoma | Survival | 82 |
| RDgCol0709A0653 | 70 | Female | 5×4.5×1.5cm | Adenocarcinoma | Death | 2 |
| RDgCol0709A0654 | 73 | Female | 3×2×1cm | Tubular adenocarcinoma | Death | 28 |
| RDgCol0709A0655 | 47 | Female | 9×4.5×1.5cm | Tubular adenocarcinoma | Survival | 81 |
| RDgCol0709A0656 | 68 | Male | 4×6×5cm | Adenocarcinoma | Survival | 81 |
| RDgCol0710A0665 | 72 | Female | 4×4×1.5cm | Tubular adenocarcinoma | Survival | 81 |
| RDgCol0710A0666 | 90 | Male | 5×2.5×1cm | Adenocarcinoma | Death | 8 |
| RDgCol0710A0667 | 65 | Male | 4×3×1.5cm | Tubular adenocarcinoma | Survival | 81 |
| RDgCol0710A0668 | 61 | Male | 7×5×1.5cm | Tubular adenocarcinoma | Survival | 81 |
| RDgCol0710A0669 | 61 | Female | 6×4.5×1.5cm | Tubular adenocarcinoma | Death | 10 |
| RDgCol0710A0663 | 78 | Female | 4×3×1.5cm | Adenocarcinoma | Death | 16 |
| RDgCol0710A0671 | 67 | Male | 5×4×1cm | Tubular adenocarcinoma | Death | 39 |
| RDgCol0710A0672 | 55 | Female | 3×2×1.8cm | Tubular adenocarcinoma | Survival | 80 |
| RDgCol0710A0673 | 68 | Male | 3.5×3×1cm | Tubular adenocarcinoma | Survival | 80 |
| RDgCol0710A0674 | 76 | Male | 6×3.5×1.5cm | Adenocarcinoma | Death | 10 |
| RDgCol0710A0675 | 85 | Male | 7×3×1.5cm | Tubular adenocarcinoma | Survival | 80 |
| RDgCol0710A0676 | 63 | Female | 6×4×1.5cm | Tubular adenocarcinoma | Survival | 80 |
| RDgCol0710A0679 | 62 | Male | 6×5×1.5cm | Tubular adenocarcinoma | Survival | 80 |
| RDgCol0710A0680 | 72 | Female | 3×3×1cm | Adenocarcinoma | Death | 9 |
| RDgCol0710A0681 | 78 | Female | 3.5×2×1cm | Adenocarcinoma | Survival | 80 |
| RDgCol0711A0771 | 63 | Male | 5×4×1cm | Adenocarcinoma | Survival | 80 |
| RDgCol0711A0775 | 62 | Male | 9×3×3cm | Tubular adenocarcinoma | Death | 16 |
| RDgCol0711A0777 | 75 | Male | 5×5×4cm | Mucinous adenocarcinoma | Survival | 79 |
| RDgCol0711A0778 | 58 | Male | 4×3×1cm | Tubular adenocarcinoma | Survival | 79 |
| RDgCol0711A0779 | 83 | Female | 5×4.5×2cm | Adenocarcinoma | Death | 5 |
| RDgCol0711A0781 | 75 | Male | 4×4×1cm | Tubular adenocarcinoma | Survival | 78 |
| RDgCol0711A0782 | 70 | Male | 6.5×4.5×1cm | Adenocarcinoma | Death | 75 |
| RDgCol0712A0825 | 51 | Male | 5×5×1cm | Tubular adenocarcinoma | Survival | 78 |
| RDgCol0712A0827 | 86 | Female | 6×4.5×1.5cm | Tubular adenocarcinoma | Survival | 78 |
| RDgCol0712A0828 | 72 | Male | 6×3×1cm | Tubular adenocarcinoma | Death | 44 |
| RDgCol0712A0829 | 65 | Male | 7×5×2cm | Tubular adenocarcinoma | Death | 29 |
| RDgCol0712A0830 | 80 | Male | 5×4×1.5cm | Tubular adenocarcinoma | Death | 11 |
| RDgCol0712A0831 | 75 | Female | 6×4×1.5cm | Adenocarcinoma | Survival | 77 |
| RDgCol0712A0833 | 60 | Female | 4×3×3cm | Mucinous adenocarcinoma | Death | 31 |
| RDgCol0712A0834 | 78 | Male | 4.5×3.5×1cm | Tubular adenocarcinoma | Survival | 77 |
| RDgCol0712A0835 | 51 | Male | 2×1.5×1cm | Adenocarcinoma | Survival | 77 |
| RDgCol0801A0880 | 72 | Male | 3×2.5×1.8cm | Tubular adenocarcinoma | Survival | 77 |
| RDgCol0801A0881 | 72 | Female | 4×3.5×2.5cm | Tubular adenocarcinoma | Death | 13 |
| RDgCol0801A0882 | 65 | Male | 5×2×1.2cm | Tubular adenocarcinoma | Death | 12 |
| RDgCol0801A0885 | 79 | Female | 4×3×1.5cm | Tubular adenocarcinoma | Survival | 77 |
| RDgCol0801A0886 | 61 | Female | 5×4×1cm | Tubular adenocarcinoma | Survival | 77 |
| RDgCol0801A0887 | 73 | Male | 7×4×1.8cm | Tubular adenocarcinoma | Death | 32 |
| RDgCol0801A0888 | 71 | Male | 5×5×0.5cm | Tubular adenocarcinoma | Survival | 76 |
| RDgCol0801A0890 | 65 | Male | 6×4.5×1.5cm | Tubular adenocarcinoma | Death | 13 |
| RDgCol0801A0891 | 52 | Female | 9×6×1.5cm | Adenocarcinoma | Death | 16 |
| RDgCol0802A0936 | 81 | Female | 4.8×3.5×1.5cm | Tubular adenocarcinoma | Survival | 76 |
| RDgCol0802A0937 | 67 | Female | 4×4×1.3cm | Tubular adenocarcinoma | Death | 36 |
| RDgCol0802A0941 | 54 | Male | 3×3×1cm | Tubular adenocarcinoma | Death | 45 |
| RDgCol0802A0943 | 60 | Male | 5×4×1cm | Adenocarcinoma | Death | 22 |
| RDgCol0802A0945 | 69 | Female | 2.8×2.5×1cm | Tubular adenocarcinoma | Survival | 75 |
| RDgCol0803A0952 | 64 | Female | 3.5×2.7×1cm | Adenocarcinoma | Survival | 74 |
| RDgCol0803A0953 | 65 | Male | 6×5×2cm | Tubular adenocarcinoma | Death | 38 |
| RDgCol0803A0954 | 83 | Male | 10×7×1.5cm | Adenocarcinoma | Death | 18 |
| RDgCol0803A0955 | 54 | Female | 3.3×2.7×1cm | Adenocarcinoma | Survival | 74 |
| RDgCol0803A0956 | 79 | Female | 5×4×1.5cm | Tubular adenocarcinoma | Survival | 74 |
| RDgCol0803A0957 | 60 | Female | 6×3.5×1.5cm | Tubular adenocarcinoma | Survival | 74 |
| RDgCol0803A0959 | 50 | Female | 3×2.5×1cm | Tubular adenocarcinoma | Survival | 74 |
| RDgCol0803A0960 | 54 | Female | 3×3×0.8cm | Tubular adenocarcinoma | Survival | 74 |
| RDgCol0803A0962 | 62 | Female | 5×3.5×1cm | Tubular adenocarcinoma | Death | 66 |
| RDgCol0803A0963 | 74 | Male | 5×4.5×1.5cm | Adenocarcinoma | Survival | 74 |
| RDgCol0803A0966 | 67 | Male | 9.5×6×2cm | Tubular adenocarcinoma | Survival | 74 |
| RDgCol0803A0967 | 65 | Female | 5×5×1.5cm | Adenocarcinoma | Death | 36 |
| RDgCol0804A0974 | 75 | Male | 3×3×1cm | Tubular adenocarcinoma | Survival | 74 |
| RDgCol0804A0977 | 70 | Male | 4.5×3×1cm | Tubular adenocarcinoma | Survival | 74 |
| RDgCol0804A0980 | 73 | Female | 6.5×5×1.5cm | Adenocarcinoma | Survival | 74 |
| RDgCol0804A0982 | 58 | Male | 6×5×1.5cm | Adenocarcinoma | Survival | 74 |
| RDgCol0804A0983 | 71 | Female | 2.5×2.5×1cm | Adenocarcinoma | Death | 32 |
| RDgCol0804A0986 | 75 | Female | 7×4×1.5cm | Mucinous adenocarcinoma | Death | 57 |
| RDgCol0804A0987 | 70 | Female | 5×4×3cm | Adenocarcinoma | Death | 51 |
| RDgCol0804A0989 | 76 | Female | 6×4.5×1cm | Signet ring adenocarcinoma | Death | 4 |
